# Post-circumcision penile epidermal inclusion cyst in an 8-year-old: A rare late complication of neonatal surgery

**DOI:** 10.1016/j.eucr.2026.103541

**Published:** 2026-07-10

**Authors:** Mulualem Amare Woldemichael, Esubalew Mihiret Alemayehu, Bedilu Zewdu Asmare

**Affiliations:** a Department of Surgery, College of Medicine and Health Science, Wolkite University, Wolkite, Ethiopia; b Department of Pediatrics, College of Medicine and Health Science, Dire Dawa University, Dire Dawa, Ethiopia; c Department of Radiology, College of Medicine and Health Science, Wolkite University, Wolkite, Ethiopia

**Keywords:** Epidermal inclusion cyst, Penile cyst, Circumcision complication, Epidermoid cyst

## Abstract

Post-circumcision penile epidermal inclusion cysts (EICs) are exceptionally rare late complications of neonatal circumcision, arising from iatrogenic implantation of squamous epithelium into subcutaneous tissue. We report an 8-year-old boy presenting with a painless penile shaft mass eight years after uneventful neonatal circumcision. Ultrasonography with color Doppler demonstrated an avascular cystic lesion without urethral or corporal communication. Complete surgical excision confirmed a benign epidermal inclusion cyst on histopathology, with no evidence of malignancy. No recurrence was observed at six-month follow-up. Clinicians should consider EICs when evaluating painless penile masses in circumcised children, regardless of the interval since surgery.

## 1 Introduction

Neonatal circumcision is one of the most commonly performed surgical procedures worldwide, with an overall complication rate of approximately 0.2–0.6%.^1^ Recognized early complications include bleeding, infection, and wound dehiscence, while late sequelae such as meatal stenosis, skin bridges, and inclusion cysts are far less frequently reported.^2^^,^^3^ Penile epidermal inclusion cysts (EICs) are an exceptionally rare subset of post-circumcision sequelae, estimated to account for less than 0.01% of all documented subcutaneous skin lesions.^4^

EICs arise when viable stratified squamous epithelial cells are iatrogenically displaced into subcutaneous tissue during surgical manipulation, subsequently proliferating within a closed space and accumulating desquamated keratin debris.^5^^,^^6^ On the penile shaft, the absence of pilosebaceous units makes post-surgical implantation the predominant pathogenic mechanism, unlike the de novo formation that characterizes scrotal or body-wall EICs.^7^ These lesions may remain clinically silent for months to decades.^8^ often leading to delayed or missed diagnosis.

We report a histopathologically confirmed EIC of the penile shaft arising eight years after uneventful neonatal circumcision and discuss the clinical, radiological, surgical, and pathological features essential for accurate diagnosis and management.

## 2 Case presentation

### 2.1 History and clinical presentation

An 8-year-old male was referred with a painless penile swelling noted by his parents over several years. The lesion had remained stable for an extended period but showed a gradual, progressive increase in size over the preceding 12 months, prompting surgical evaluation. He had undergone routine neonatal circumcision on the seventh day of life without documented intraoperative or postoperative complications. There was no history of penile trauma, local infections, or prior interventions. The patient was entirely asymptomatic with respect to urinary function, with no dysuria, hematuria, or voiding dysfunction.

### 2.2 Physical examination

**Fig. 1:**
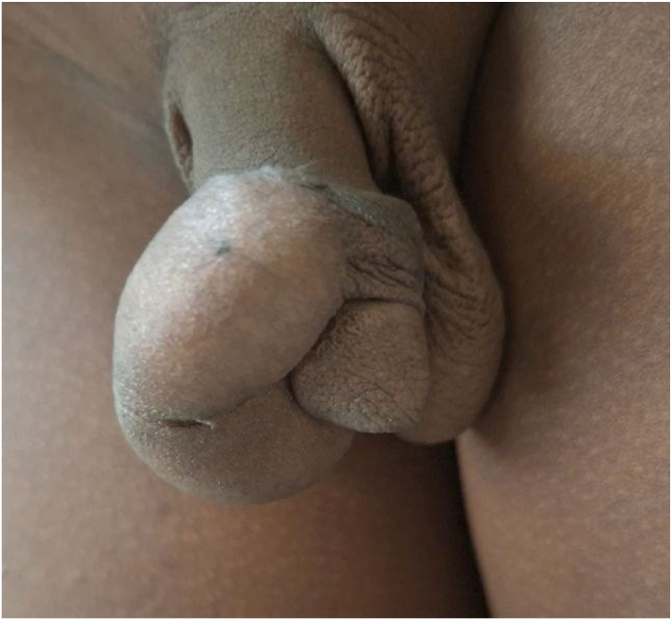
Pre-operative clinical photograph demonstrating the well-circumscribed, dome-shaped cystic swelling on the dorsolateral aspect of the distal penile shaft. Overlying skin is intact with no inflammatory changes, punctum, or sinus tract.

General examination was unremarkable for his age. Focused genital examination revealed a well-circumscribed, smooth-surfaced, dome-shaped cystic mass measuring approximately 1.5 × 1.0 × 1.0 cm on the dorsolateral aspect of the distal penile shaft, within the subcutaneous tissue beneath intact overlying skin. The overlying skin showed no erythema, sinus tracts, puncta, or scarring. The mass was soft, non-tender, fluctuant, and freely mobile over the underlying corpora cavernosa. The urethra was clearly uninvolved. There was no inguinal lymphadenopathy, and both testes were fully descended (Fig. 1).

### 2.3 Investigations and imaging

**Fig. 2:**
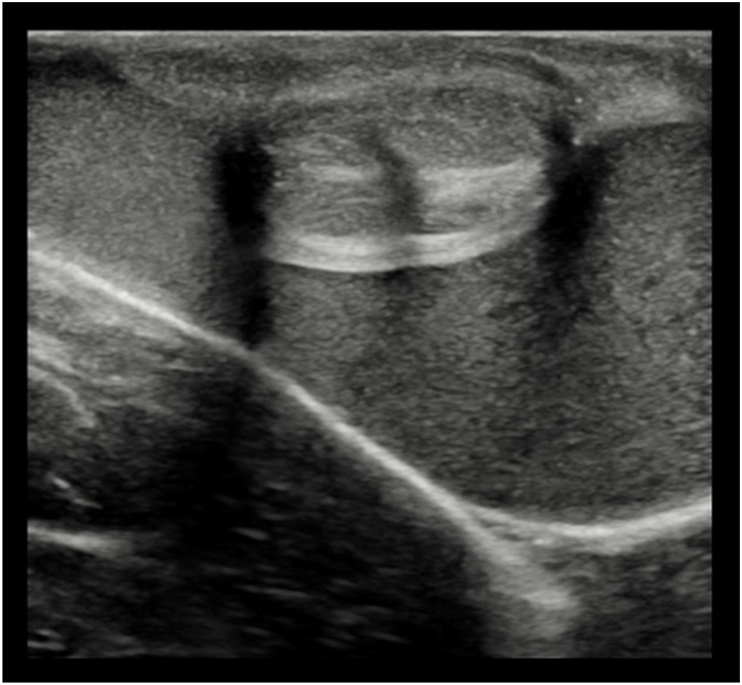
Longitudinal ultrasound of the penile shaft demonstrating a well-defined subcutaneous cystic lesion along the dorsolateral aspect (arrow). The lesion is hypoechoic with internal echogenic debris and posterior acoustic enhancement. No internal vascularity on color Doppler. The lesion is clearly separated from the corpora cavernosa and corpus spongiosum (asterisk), favoring epidermoid cyst.

Routine hematological and biochemical investigations were within normal limits. High-resolution B-mode ultrasonography revealed a well-defined, thin-walled, hypoechoic to anechoic cystic structure within the subcutaneous tissue superficial to Buck's fascia on the dorsolateral penile shaft, without internal septations, calcifications, or echogenic debris. Color Doppler confirmed a complete absence of internal vascularity and no communication between the cyst and the urethral lumen or corpora cavernosa, effectively excluding urethral diverticulum and paraurethral cyst (Fig. 2).

### 2.4 Surgical management

**Fig. 3:**
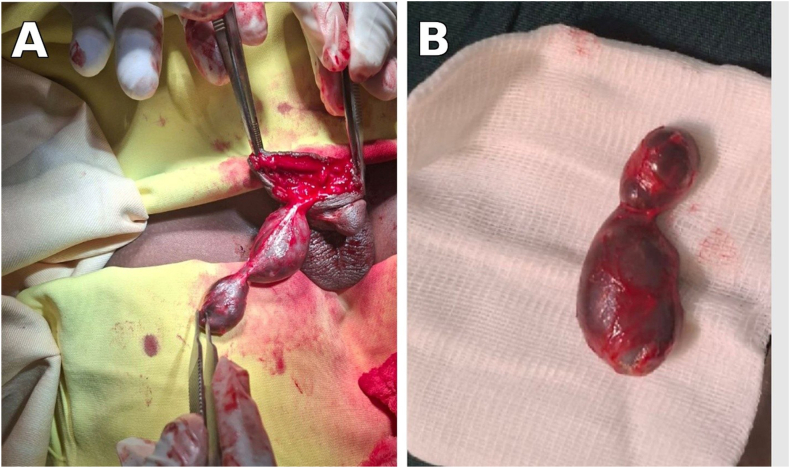
(A) Intraoperative photograph demonstrating surgical dissection and delivery of the intact cyst from the subcutaneous plane superficial to Buck's fascia. The well-encapsulated, glistening surface of the cyst is visible. (B) Gross specimen photograph of the excised cyst showing a bilobed configuration (approximately 20 mm), intact smooth external surface, and dark-red post-excision coloration.

Elective surgical excision was performed under general anesthesia. Following standard sterile preparation and draping, a fusiform incision was made over the longitudinal axis of the mass. Careful dissection in the subcutaneous plane immediately superficial to Buck's fascia allowed complete en-bloc excision without intraoperative rupture of the cyst wall, preventing spillage of keratinaceous contents and reducing the risk of implantation recurrence. The excision site was closed in layers using absorbable sutures. Hemostasis was secured throughout (Fig. 3).

### 2.5 Histopathological examination

**Fig. 4:**
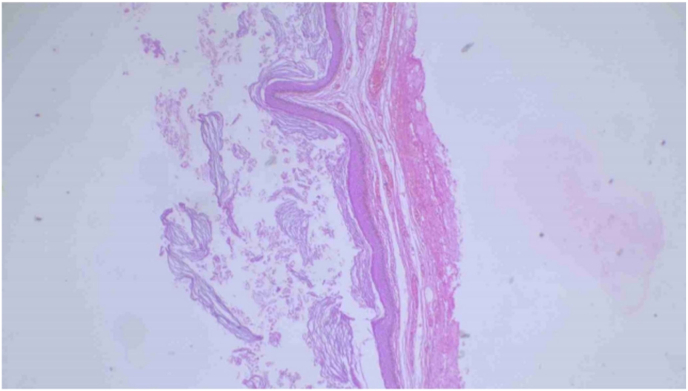
Photomicrograph (H&E stain) demonstrating the cyst wall lined by stratified squamous epithelium with a granular layer; the lumen is filled with laminated keratinaceous material. Negative for malignancy.

Gross macroscopic examination confirmed a white-tan-brown soft tissue specimen measuring 20 mm. Microscopic evaluation with hematoxylin and eosin (H&E) staining demonstrated a cyst lined by stratified squamous epithelium with a prominent granular cell layer, its lumen filled with concentric laminated layers of anucleate keratinaceous material. No hair follicles, sebaceous glands, or eccrine sweat glands were identified within the cyst wall, confirming classification as an epidermal inclusion cyst rather than a dermoid cyst. Sections were negative for dysplasia, atypia, and malignancy. The pathological diagnosis was: Benign Epidermoid Cyst, Penis — Excision (Fig. 4)

### 2.6 Postoperative course and follow-up

**Fig. 5:**
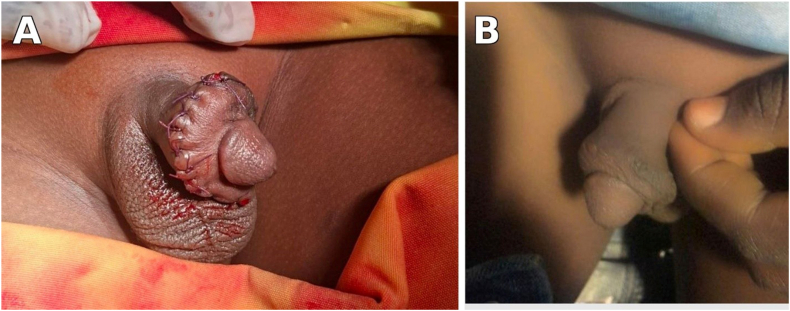
(A) Post-operative appearance following wound closure with absorbable sutures. (B) Six-month postoperative photograph demonstrating complete wound healing and no evidence of recurrence.

The patient was discharged on the same day without complications. At the six-month postoperative follow-up, the surgical site was well-healed with a satisfactory cosmetic result. There was no clinical evidence of local recurrence, residual cystic lesion, or distortion of penile architecture, and urinary function remained entirely normal throughout the follow-up period (Fig. 5).

## 3 Discussion

Penile EICs following neonatal circumcision are genuinely rare, with the literature largely consisting of isolated case reports or small series ^1^^,^^2^^,^^4^, ^5^, ^6^, ^7^, ^8^, ^9^. Their true incidence remains unquantified, partly due to chronic underreporting of minor late complications of a procedure often performed outside specialist surgical settings.^2^

The established pathogenesis rests on iatrogenic epidermal cell implantation: minute fragments of viable stratified squamous epithelium become inadvertently buried in subcutaneous tissue during wound closure, subsequently proliferating and desquamating within an enclosed space to form a progressive keratin-filled cyst.^5^^,^^6^^,^^9^ The dorsolateral location in our case corresponded precisely to the expected circumcision line, strongly supporting this mechanism.

The highly variable and often prolonged latency between the inciting event and clinical presentation is a cardinal feature. Published cases describe presentation from months to decades after circumcision.^8^^,^^9^ a 2-year latency in a 4-year-old.^6^ a 9-year latency in a 10-year-old,^8^ and adult presentations.^9^ Our patient's 8-year latency is consistent with this pattern and underscores the importance of obtaining a complete surgical history when evaluating any penile mass, regardless of age or interval since surgery.

The clinical differential diagnosis includes median raphe cyst, dermoid cyst, mucoid cyst, urethral diverticulum, paraurethral cyst, and mesenchymal tumors. High-resolution ultrasonography with color Doppler is the non-invasive modality of choice to characterize the lesion and exclude these alternatives.^4^^,^^7^^,^^10^ The anechoic appearance, absent vascularity, and lack of urethral or corporal communication in this case were all consistent with a benign cystic etiology.

Complete surgical excision is the definitive treatment, providing histopathological confirmation and preventing late complications including progressive enlargement, secondary infection, spontaneous rupture with foreign-body granulomatous reaction, intracystic keratin calculus formation, and fistulation.^9^ Preservation of cyst wall integrity during dissection is technically paramount, as intraoperative rupture increases the risk of local recurrence from residual viable epithelial cells.^4^^,^^6^ Although malignant transformation of penile EICs has not been definitively documented, histopathological examination after excision remains mandatory to exclude unexpected or atypical pathology.

## 4 Conclusion

Post-circumcision penile EICs are rare but clinically significant late complications that may present years to decades after an uneventful neonatal procedure. Clinicians across specialties must maintain a high index of suspicion for EICs when evaluating painless, slowly progressive penile shaft masses in children with a prior circumcision history, regardless of the time elapsed. High-resolution ultrasonography with color Doppler effectively characterizes the lesion and excludes relevant differential diagnoses. Complete surgical excision with histopathological confirmation yields excellent functional and cosmetic outcomes and remains the standard of care.

## Availability of data and materials

All relevant data supporting the findings of this study are included within the manuscript.

## Ethics approval and consent to participate

Ethical clearance was not required for this case report per institutional policy. Written informed consent was obtained from the patient's parent/legal guardian for publication of this case report and accompanying images.

## Consent for publication

Written informed consent for publication of clinical details and accompanying images was obtained from the patient's parent/legal guardian.

## Funding

The authors received no financial support for the research, authorship, or publication of this article.

## CRediT authorship contribution statement

**Mulualem Amare Woldemichael:** Conceptualization, Writing – original draft. **Esubalew Mihiret Alemayehu:** Writing – review & editing. **Bedilu Zewdu Asmare:** Validation, Visualization.

## Competing interests

The authors declare no conflict of interest.
